# Prevalence of Lymphatic Filariasis and Treatment Effectiveness of Albendazole/ Ivermectin in Individuals with HIV Co-infection in Southwest-Tanzania

**DOI:** 10.1371/journal.pntd.0004618

**Published:** 2016-04-12

**Authors:** Inge Kroidl, Elmar Saathof, Lucas Maganga, Petra Clowes, Leonard Maboko, Achim Hoerauf, Williams H. Makunde, Antelmo Haule, Prisca Mviombo, Bettina Pitter, Neema Mgeni, Joseph Mabuye, Dickens Kowuor, Upendo Mwingira, Mwelecele N. Malecela, Thomas Löscher, Michael Hoelscher

**Affiliations:** 1 Division of Infectious Diseases and Tropical Medicine, Medical Center of the University of Munich (LMU), Munich, Germany; 2 National Institute of Medical Research (NIMR)-Mbeya Medical Research Centre (MMRC), Mbeya, Tanzania; 3 German Center for Infection Research (DZIF), Munich, Germany; 4 German Center for Infection Research (DZIF), Bonn-Cologne, Germany; 5 Institute of Medical Microbiology, Immunology and Parasitology, Bonn, Germany; 6 National Institute of Medical Research (NIMR), Tanga, Tanzania; 7 National Institute of Medical Research (NIMR), Dar es Salaam, Tanzania; Institute of Tropical Medicine, BELGIUM

## Abstract

**Background:**

Annual mass treatment with ivermectin and albendazole is used to treat lymphatic filariasis in many African countries, including Tanzania. In areas where both diseases occur, it is unclear whether HIV co-infection reduces treatment success.

**Methodology:**

In a general population study in Southwest Tanzania, individuals were tested for HIV and circulating filarial antigen, an indicator of *Wuchereria bancrofti* adult worm burden, before the first and after 2 consecutive rounds of anti-filarial mass drug administration.

**Principle Findings:**

Testing of 2104 individuals aged 0–94 years before anti-filarial treatment revealed a prevalence of 24.8% for lymphatic filariasis and an HIV-prevalence of 8.9%. Lymphatic filariasis was rare in children, but prevalence increased in individuals above 10 years, whereas a strong increase in HIV was only seen above 18 years of age. The prevalence of lymphatic filariasis in adults above 18 years was 42.6% and 41.7% (p = 0.834) in HIV-negatives and–positives, respectively. Similarly, the HIV prevalence in the lymphatic filariasis infected (16.6%) and uninfected adult population (17.1%) was nearly the same. Of the above 2104 individuals 798 were re-tested after 2 rounds of antifilarial treatment. A significant reduction in the prevalence of circulating filarial antigen from 21.6% to 19.7% was found after treatment (relative drop of 8.8%, McNemar´s exact p = 0.036). Furthermore, the post-treatment reduction of CFA positivity was (non-significantly) larger in HIV-positives than in HIV-negatives (univariable linear regression p = 0.154).

**Conclusion/Significance:**

In an area with a high prevalence for both diseases, no difference was found between HIV-infected and uninfected individuals regarding the initial prevalence of lymphatic filariasis. A moderate but significant reduction in lymphatic filariasis prevalence and worm burden was demonstrated after two rounds of treatment with albendazole and ivermectin. Treatment effects were more pronounced in the HIV co-infected subgroup, indicating that the effectiveness of antifilarial treatment was not reduced by concomitant HIV-infection. Studies with longer follow-up time could validate the observed differences in treatment effectiveness.

## Author Summary

Parasite infections and HIV show large geographical overlap in sub-Saharan Africa and could hence potentially interact in co-infected individuals. In a general-population study conducted in Southwest Tanzania, we found high prevalence of both, lymphatic filariasis and HIV, with 42.5% of the adult population infected with *Wuchereria bancrofti* and 16.8% infected with HIV. Seven percent of the adults were infected with both pathogens. When adjusting for age, there was no statistically significant difference in initial prevalence or worm burden between HIV-positive and negative participants. For 798 individuals test results for both diseases were available in 2009, before and in 2011, after 2 rounds of treatment against lymphatic filariasis. Between 2009 and 2011, a significant drop of prevalence and worm burden in infected individuals were observed, which was more pronounced in the HIV co-infected subgroup. Hence, HIV co-infection does not seem to negatively affect lymphatic filariasis treatment programmes.

## Introduction

Lymphatic Filariasis (LF) is a mosquito-borne disease caused either by *Wuchereria bancrofti* which is distributed throughout the tropics, or *Brugia malayi and Brugia timori*, both limited to Southeast-Asia. It is estimated that 120 million people world-wide are infected with one of these pathogens, and 1 billion are at risk to acquire LF during their lifetime [1]. Before larger treatment programmes started, LF was present in most of the 21 regions of Tanzania with up to 63.8% of individuals testing positive for circulating filarial antigen, a marker for LF infection [2]. Since the year 2000 the “Global Alliance to Eliminate Lymphatic Filariasis” uses annual mass drug administration (MDA), with the aim to control and ultimately eliminate the disease [3]. The campaign of the Tanzanian National Lymphatic Filariasis Elimination Programme (NLEFP) commenced in 2001 in the coastal regions of Tanzania. In the Mbeya district in Southwest-Tanzania the treatment programme started in October 2009 with the annual distribution of albendazole (400mg) and ivermectin (150–200μg/kg). Ivermectin is considered to be mainly microfilaricidal [4], for albendazole an effect on the release of intrauterine antigen components of the adult worm was described [5]. Some studies report on the treatment effectiveness of the combination of albendazole and ivermectin after 12-month: in Ghana a significant reduction in circulating filarial antigen (CFA) levels but no measurable reduction of CFA prevalence was described in 370 individuals receiving both drugs [6, 7]. A longitudinal study from Northern Tanzania showed only small reductions of CFA positivity after two annual drug distributions (from 53.3% to 51.4%), but a significant drop to 44.9% and 19.6% after four and seven years of treatment, respectively [8].

In South Western Tanzania, both LF and HIV are public health concerns. The HIV prevalence in the country has been documented in several national surveys [1, 9, 10]. The third population based Tanzanian HIV/AIDS and Malaria Indicator Survey in 20011/2012 (THMIS) revealed a country-wide HIV prevalence of 5.1% in Tanzanian adults between the age of 15 and 49 years, and a prevalence of 9.0% for this age-group in Mbeya Region [10]. Large scale distribution of antiretroviral (ART) drugs was initiated in Tanzania in 2005. At the time of our study, ART was not widely available in Southwest Tanzania. [10–12].

Local differences in initial prevalence, coverage of treatment programs, co-infection with other pathogens, etc. can all affect treatment success, thus careful surveillance of the programs is necessary to control the infection. [1, 13–17]. Only few manuscripts focus specifically on the possible interaction of HIV with LF and most of these use cross-sectional data [18–21]. Only one recently published study investigates the treatment effectiveness of MDA drugs in HIV/LF co-infected individuals [22], but focusses on changes in CD4 and HIV viral load after antifilarial treatment in selected HIV-positive individuals. No study concentrated on the antifilarial treatment effectiveness of MDA drugs in HIV/LF co-infected individuals.

Our study assesses LF prevalence in the Mbeya Region, before and after the governmental eradication program reached the area and examines the potential impact of HIV co-infection on LF treatment.

## Methods

### Study population and study design

**Fig 1:**
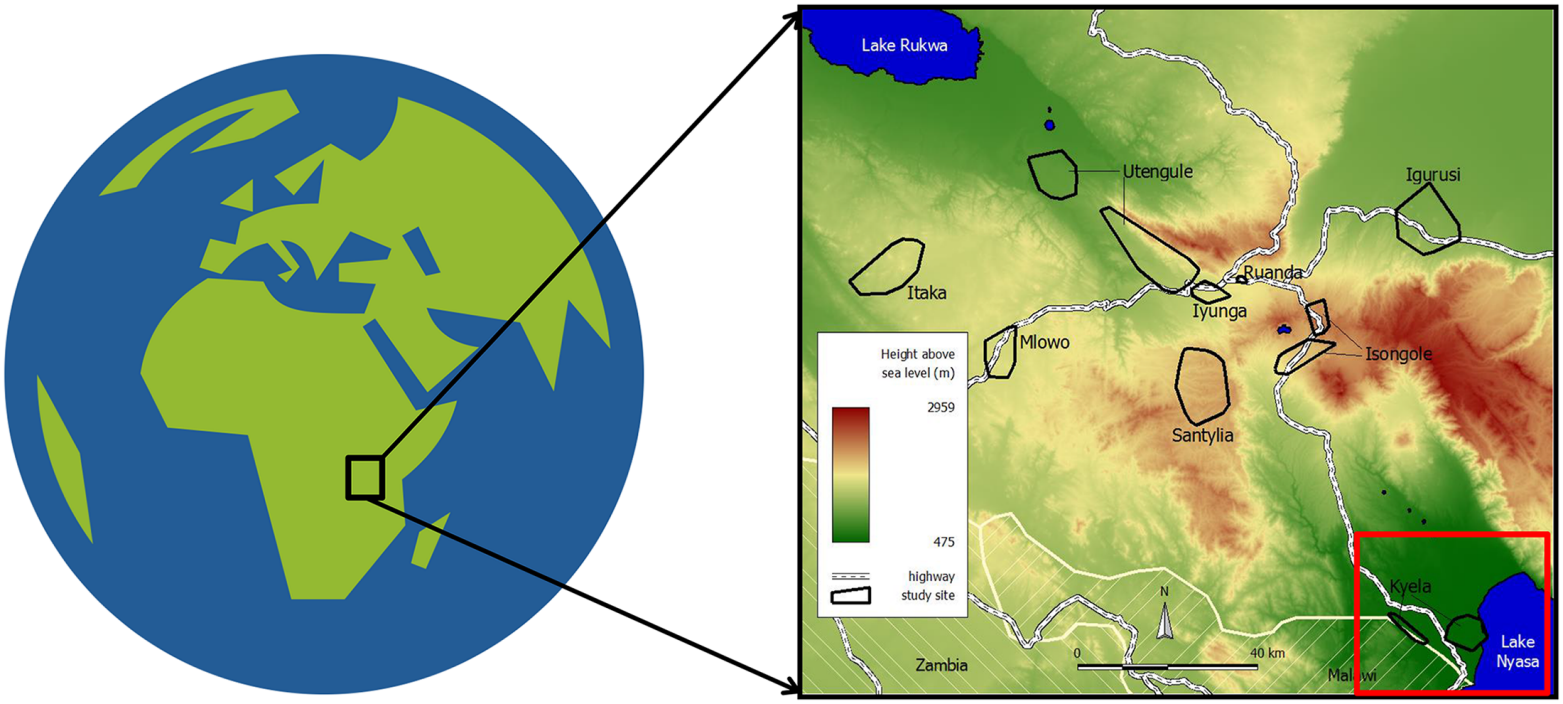
Study area. The study was conducted in the southwestern part of Tanzania (black rectangle). A general population study was performed in nine study areas (black polygons). Data for this study were collected in Kyela (red rectangle) situated close to Lake Nyassa. (Globe Icon by Sev, https://openclipart.org).

Data were collected during the SOLF cohort-study (**S**urveillance **o**f **L**ymphatic **F**ilariasis, http://www.mmrp.org/projects/basic-research/solf.html) in the Kyela district/Mbeya region in Southwest Tanzania which was conducted at the National Institute for Medical Research (NIMR)—Mbeya Medical Research Centre (MMRC) between 2009 and 2011. The study was embedded into the population based EMINI (**E**valuation and **M**onitoring of the **I**mpact of **N**ew **I**nterventions, http://www.mmrp.org/projects/cohort-studies/emini.html) cohort study, which was carried out in 9 selected communities in the Mbeya region (Fig 1) from 2006 to 2011. More than 170,000 inhabitants from ~42,000 households of these communities were registered and 10% of households randomly selected to participate in the study. No additional households entered the surveillance, but some new participants entered through birth or marriage into included household.

### Ethical considerations

The SOLF study was approved by the Mbeya Medical Research and Ethics Committee and the Tanzanian National Institute for Medical Research—Medical Research Coordinating Committee as an amendment to the EMINI cohort study. Prior to enrolment, each EMINI participant had provided written informed consent regarding study participation. Parents consented for their children below 18 years of age. In addition, children above the age of 12 years signed their own assent form.

### Data collection

Data and samples from participants in the Kyela site of the EMINI study were collected annually from 2007 until 2009. During the last two surveys (2010 and 2011) only half of the study households were visited in each year. During each visit, which took place between 8 am and 2 pm, blood, urine and stool samples were collected from each participant. Samples from 2,165 participants from March 2009 were used to estimate the prevalence of LF directly before the government treatment program commenced in Kyela in October 2009. In March 2011, 18 month after the first and 6 month after the second delivery of antifilarial treatment, samples from 1,010 participants were used to evaluate treatment impact.

### Sample processing

From each study participant, 2.7 ml of blood was collected during morning hours in EDTA tubes and immediately stored at 4°C. Cells and plasma were separated within 24 hours and subsequently stored at -80°C. All laboratory tests were performed at NIMR-MMRC, Mbeya Tanzania.

### HIV diagnosis

HIV testing was performed using the SD-Bioline HIV-1/2 3.0 (Standard Diagnostics, Kyonggi-do, South Korea) rapid diagnostic test (RDT). Negative RDT results from one survey, followed by another negative RDT result in a subsequent survey, were regarded as confirmed negative and not further tested. All positive results were confirmed using an ELISA HIV test (Enzygnost Anti HIV 1/2 Plus, DADE-Behring, Marburg, Germany), and tested by Western blot (MPD HIV Blot 2.2, MP Biomedicals, Geneva, Switzerland) if discordant. For all HIV incident cases, the negative result of the previous year, as well as the new positive results was confirmed by the testing algorithm described above. For children below the age of two years, HIV testing was done by PCR. Further details are described elsewhere [23]. Because confidential disclosure of the HIV-status could not be ensured during household visits, we did not inform participants about their HIV status. Instead they were offered voluntary counseling and testing by an independent team, which was travelling with our study team, who provided referral to the local care and treatment center, to everyone who was tested positive.

### Filarial antigen testing

A commercially available ELISA (TropBio Og4C3 serum ELISA, Townsville, Australia) was used to detect circulating filarial antigen (CFA) using 100 μl of the collected sera. The Og4C3 antibody detects *Wuchereria bancrofti* antigen with high specificity (98.5%) and no known cross-reaction to *Onchocerca volvulus*, *Brugia spp*., *Mansonella*, *Dracunculus medinensis*, *Ascaris lumbricoides* or *Strongyloides stercoralis* [24]. Sensitivity varies between 73% [25] and 100% [26], but was found 97.9% in individuals carrying microfilariae [24]. CFA is secreted by fully developed *W*. *bancrofti* adults and can be found at similar levels during day and night. Antigen levels thus reflect the *W*. *bancrofti* worm burden. The measurement of CFA with the Trop Bio ELISA is semi-quantitative; seven control tubes with standardized amounts of antigen are supplied and allow an estimation of the filarial antigen levels in the analysed plasma according to the measured optical density (OD). LF test results were considered negative, indeterminate or positive if the OD was <0.2, ≥0.2 and ≤0.3, or >0.3 respectively.

### Statistics

Statistical analyses were performed using Stata statistics software (version 14; Stata Corp., College Station, TX). Pearson´s chi-squared test was used to compare binominal outcomes between groups and to compare CFA positivity before and after treatment in all participants. McNemar´s exact test for paired data was used to compare CFA positivity before and after treatment in those individuals who participated in both surveys. The non-parametric Wilcoxon rank sum test was used to compare selected baseline characteristics of continuous variables, since none of these was normally distributed. In order to examine the association of LF infection with HIV status and other potentially important covariates we performed uni- and multi-variable log link binomial regression analyses with robust variance estimates.

## Results

**Fig 2:**
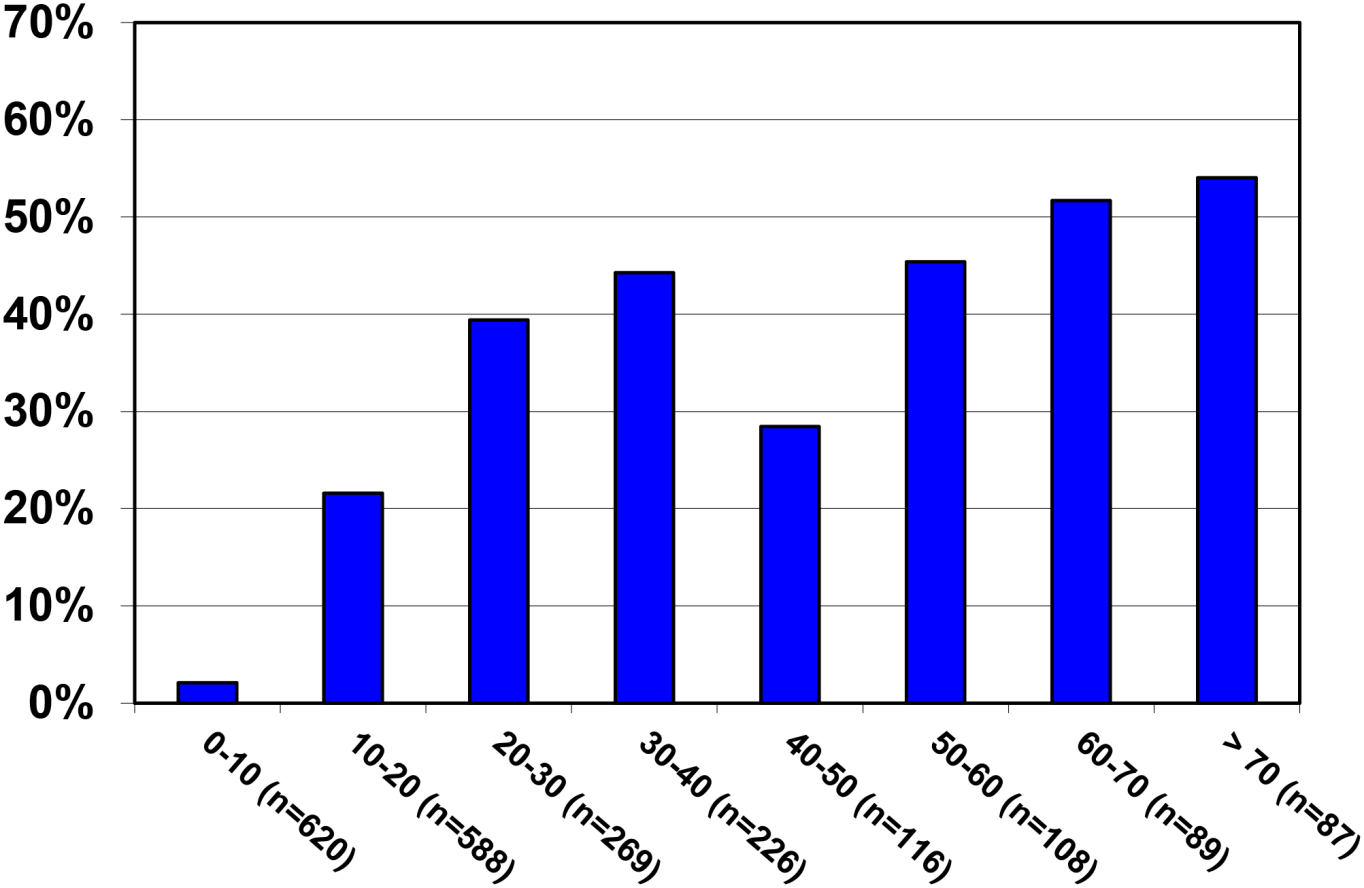
Baseline CFA prevalence by age. Numbers of participants for each age group are given in parenthesis.

In March 2009, before the first national MDA commenced, valid CFA results were obtained from 2,104 individuals (Table 1). Indeterminate results were found for the 61 of the tested 2,165 samples (2.8%). Their median age was 16.6 years (range 0–94, IQR: 8.8 to 34), and 51.0% were female. Only 4 (1.6%) of the 245 children below the age of 5 years were CFA-positive; LF prevalence started to rise in participants above 10 years and was 42.3% in adults above 18 years of age (Fig 2). When including all age groups, 24.8% of the study population were CFA-positive with a trend to higher prevalence in males (26.5%) than in females (23.1%, chi-squared p = 0.074). In the adult population above 18 years the difference in CFA-positivity between males (47.3%) and females (38.0%) was significant (chi-squared p = 0.003).

**Table 1:** Study participants and LF prevalence in 2009 and 2011.

		2009			2011	
	all n	LF-positive n	LF-prevalence %	all n	LF-positive n	LF-prevalence %
open cohort						
all*	2,104	521	24.8	974	192	19.7
HIV-negative	1,905	450	23.6	878	166	18.9
HIV-positive	187	69	36.9	91	25	27.5
closed cohort						
all**	798	172	21.6	798	157	19.7
HIV-negative	723	151	20.9	723	140	19.4
HIV-positive	69	19	27.5	69	15	21.7

*12 individuals without valid HIV result

**6 individuals without valid HIV result

Data for all study participants from 2009 and 2011 are shown in the upper part of the table (= open cohort). Only 798 individuals participated in both years of the surveillance (= closed cohort) their data are shown in the lower part.

**Fig 3:**
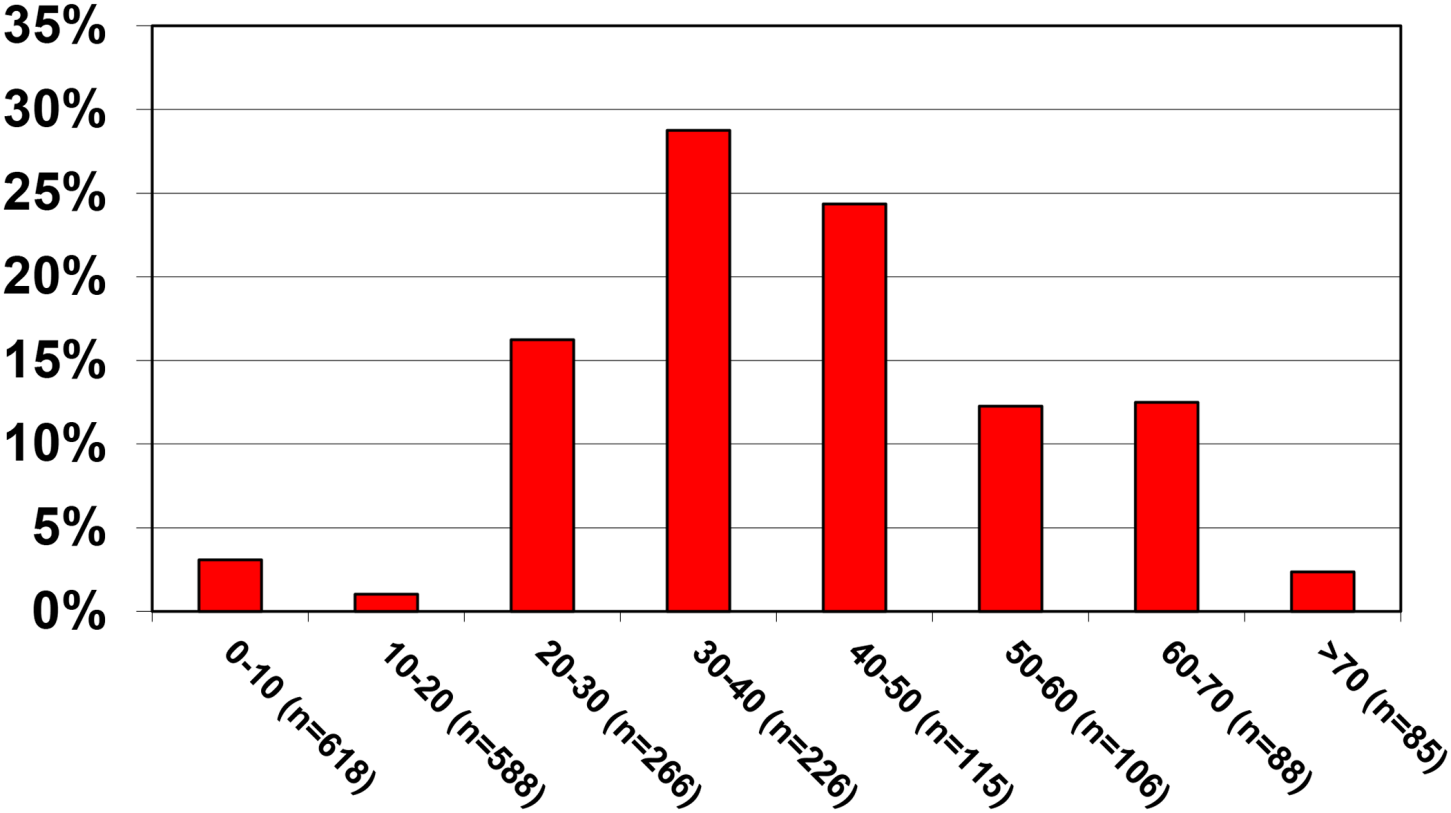
HIV prevalence by age. Numbers of participants for each age group are given in parenthesis (for 12 individuals no valid HIV result was available).

In March 2011, 18 months after the first MDA and six months after the second, ~50% of the initially included households were revisited for interviews and blood sample collection. Some scheduled participants were not found in 2011, and some new individuals had entered the visited households (see study population and design). In addition to an analysis where the data of all participants form each Survey (= open cohort) are evaluated, which reflects more a cross sectional design, a second analysis included only the 798 individuals who actively participated in both years of the surveillance longitudinally (= closed cohort). The numbers of participants is shown in Table 1. Of the 974 valid test results in 2011, 19.7% were CFA-positive, leading to a calculated prevalence reduction of 5.1% (24.8% vs. 19.7%, chi-squared p = 0.002) when including all subjects who participated in at least one survey (Table 1, open cohort). In the analysis of samples from 798 individuals who actively participated in both surveys (Table 1, closed cohort), a lower prevalence reduction (21.6 to 19.7%, McNemar´s exact p = 0.036) was measured (Fig 3).

At baseline the overall HIV prevalence in our study cohort was 8.9%, with a prevalence of only 2.1% in children and adolescents below the age of 18 years, and a prevalence of 16.9% in individuals ≥18 years of age (Fig 3).

**Fig 4:**
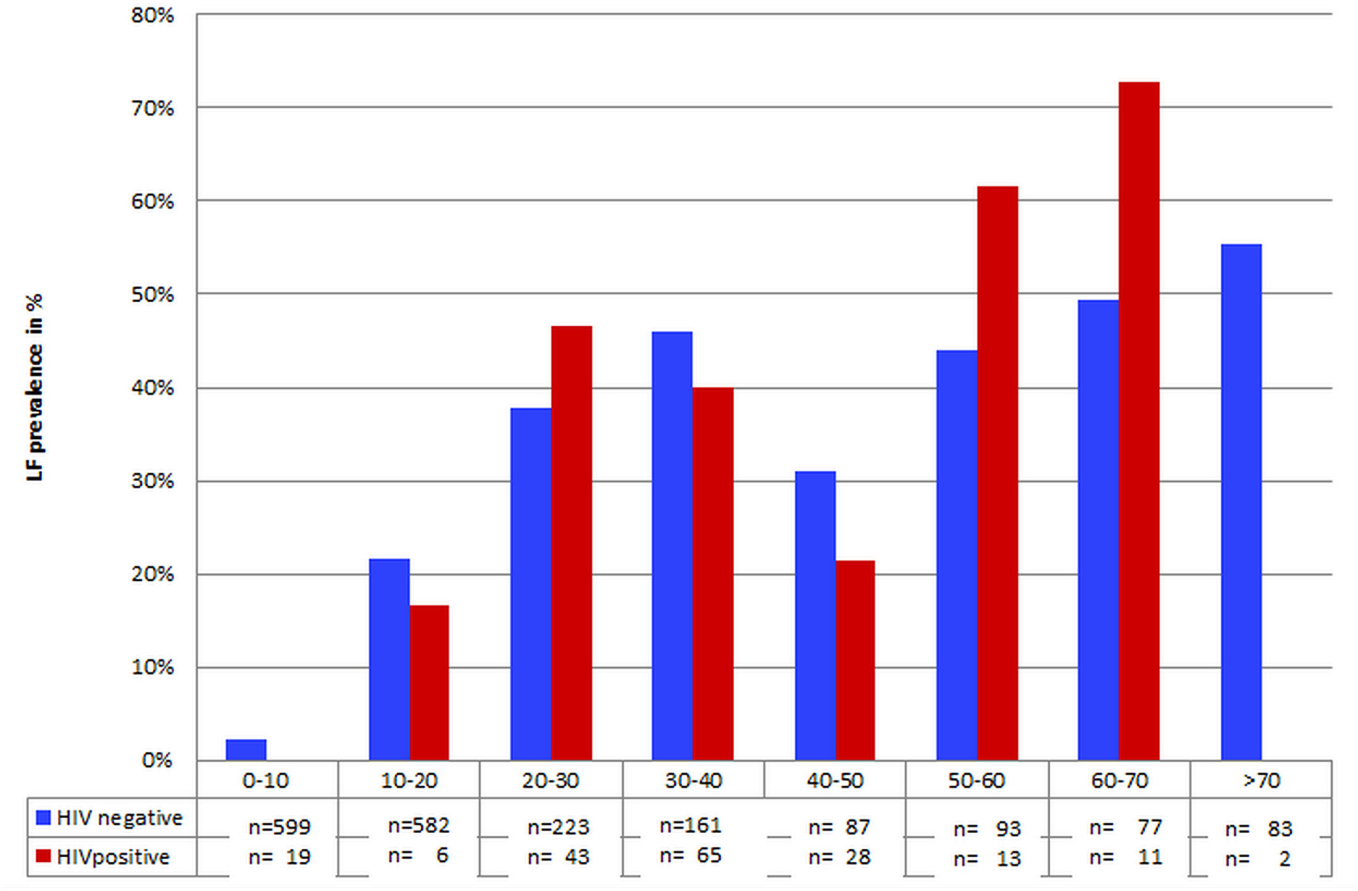
Baseline CFA prevalence by age for HIV-positive and HIV-negative participants. Prevalence of CFA positivity in HIV-negative (blue) and HIV-positive (red) participants. No HIV/LF co-infection was observed in participants below 10 years of age.

HIV-infection was more prevalent in female (10.7%), compared to male participants (7.1%, chi-squared p = 0.003). Sixty-eight of the 968 adult individuals (7.0%) were infected with both pathogens and among the whole group of 2,104 individuals 69 co-infections (= 3.3%) were observed. The initial univariable analysis of the potential association of HIV with LF infection showed a higher prevalence of LF in HIV-positive (36.9%); compared to HIV-negative individuals (23.6%) (RR = 1.56, 95% CI = 1.26 to 1.94, p<0.001). But we already demonstrated that HIV and LF are both less common in children than in adults, which confounds this association. To further study the pattern of co-infection we analysed CFA positivity in HIV infected and uninfected individuals stratified by age (Fig 4); in adults (> = 18 years) only; and in log-link binomial multivariable regression adjusted for age and gender. None of these analyses showed a significant association of LF infection with HIV, neither within the single age strata nor overall in the multivariable regression model where the influence of age and gender were confirmed, but where the adjusted RR for HIV was only 1.04 (Table 2). When only analysing data from adults above 18, the CFA prevalence was 42.6% in the HIV-negative and 41.7% in the HIV-positive subgroup (univariable log-link regression RR = 0.98, 95% CI = 0.80 to 1.20; p = 0.84).

**Table 2:** Uni- and Multivariable binomial log-link regression models showing associations of Age, Sex and HIV positivity with LF infection in 2092 individuals before antifilarial treatment commenced.

		Univariable				Multivariable		
All:	n	LF pos (%)	RR	95% CI	P	RR	95% CI	P
	2,092	24.8						
**HIV**								
**neg**	1,905	23.6	1	1.26 to 1.94	<0.001	1	0.85 to 1.27	0.689
**pos**	187	36.9	1.56			1.04		
**Sex:**								
**female**	1,067	23.2	1	0.99 to 1.32	0.064	1	1.11 to 1.44	<0.001
**male**	1,025	26.5	0.15			0.27		
**Age (years)**								
**0-<18**	1,124	9.6	1			1		
***18-<50***	689	39.2	4.01	3.27 to 5.08	<0.001	4.10	3.29 to 5.10	<0.001
**> = 50**	279	50.5	5.26	4.20 to 6.58	<0.001	4.16	4.32 to 6.70	<0.001

In order to compare antifilarial treatment success in the HIV-negative and positive subgroups we again performed two analysis: one for all tested individuals who participated in at least one survey (open cohort using chi-squared testing), and one only for the individuals who participated in both surveys before and after treatment (closed cohort, using McNemar´s exact test).

For the open cohort a CFA prevalence reduction from 23.6% to 18.9% (chi-squared p = 0.015, relative drop = 19.7%) was found in HIV-negative participants, and from 36.9% to 27.5% (chi-squared p = 0.023, relative drop = 25.4%) in HIV-positives. For the closed cohort we observed a drop in CFA positivity from 20.9% to 19.4% (McNemar´s exact p = 0.117, relative drop = 7.3%) in 723 HIV-negatives and from 27.5% to 21.7% (McNemar´s exact p = 0.125, relative drop = 21.1%) in 69 HIV-positive participants. The reason for this pronounced difference (7.3% vs. 21.1%) is a higher incidence of CFA positivity in the HIV negative participants where 15 (2.6%) of the 572 initially CFA negative participants turned CFA-positive, whereas none of the 50 HIV-positive participants who were initially CFA-negative turned CFA-positive (chi-squared p = 0.246). The proportion of initially CFA positives who turned CFA negative was very similar in HIV-negative (26/151 = 17.2%) and HIV-positive participants (4/19 = 21.1%, chi-squared p = 0.679). When combining this information about change in LF status in one outcome variable (-1 = turned CFA negative; 0 = no change in CFA status; 1 = turned CFA positive) univariable linear regression modelling resulted in a coefficient β for the HIV infected subgroup of -0.043 (95%CI = -0.102 to 0.016, p = 0.154).

Analysing the prevalence reduction in the closed cohort for adults > = 18 years only, a drop from 42.7% to 40.3% (McNemar´s exact p = 0.248, relative drop = 5.6%) was noted for HIV-negatives, and from 32.7% to 25.5% (McNemar´s exact p = 0.125, relative drop = 22.0%) in the HIV-positive subgroup. Summarizing our results, we found more pronounced drops in prevalence among the HIV positive subgroup, compared with the HIV negative, no matter, whether all participants or only adults are analysed and also with both possible ways of evaluating the data (open cohort or closed cohort).

The measurement of CFA with the Trop Bio ELISA is semi-quantitative; with the OD of the plasma samples reflecting the participant’s worm burden. Our findings for CFA intensities parallel those for CFA prevalence: geometric mean intensities before treatment were relatively similar between HIV-positives (157 units) and HIV-negatives (179 units, Wilcoxon rank sum p = 0.34), which is also true for the relative reduction of geometric mean intensity after treatment, which was 26% and 30% respectively (Wilcoxon rank sum p = 0.50)

## Discussion

In our study we found a significant decrease in LF-prevalence after only 2 years of MDA in an area with high HIV co-infection in South-West Tanzania. This was in contrast to our expectations and previously published manuscripts [2, 6, 7, 16, 27]. Furthermore we did not find any evidence that HIV co-infection impairs the effectiveness of antifilarial treatment. On the contrary, our data show a more pronounced decrease in prevalence and CFA intensity among HIV-positive compared to HIV-negative participants. We tried to consider several factors which could have affected our analysis. The age distribution of LF and HIV infection had to be taken into account, but also the composition of the study population and potential changes during follow-up. However, an almost three-fold relative drop in LF prevalence was seen for HIV-positive compared to HIV-negative participants in the most stringent analysis which only considered individuals for whom we have data both before and after treatment. We do not want to overstate this result since the overall numbers of participants with HIV/LF-co-infection was low, despite an initially large cohort and accordingly the differences in cure and incidence rate between HIV-infected and uninfected participants are not significant. Moreover, we are unable to present an explanation for this finding. Reports about the LF prevalence in HIV-negative and HIV-positive individuals have been rare and conflicting in the past. Nielsen et al. described a positive association of HIV and LF infection after adjusting for age and sex in a cross-sectional study from Northern Tanzania [20], even though a further evaluation of this group of individuals did not support an association between HIV and LF [19]. No difference regarding CFA levels was found in other cross sectional studies from India [18] and Malawi [28]. The latter findings are supported by the cross sectional analysis of our study participants in 2009. To date, no other longitudinal study has compared the effectiveness of antifilarial treatment in HIV-positive and negative subgroups.

One interesting finding is the higher pre-treatment CFA prevalence in the open compared to the closed cohort which demonstrates that LF-positive individuals were more likely to be lost to follow-up than LF-negative participants. This can at least partly be explained by the higher prevalence of LF in adults, who are more likely than younger participants to relocate (e.g. in search of a job), to harbour diseases that prevent them from further study participation (e.g. HIV-infection) and to die.

Most other studies analyse the treatment effectiveness after 6, 8 or even more years of treatment. Thus one limitation of our study is the short duration of follow-up, which is a consequence of funding restrictions and does not allow for a conclusive analysis of MDA effectiveness. Furthermore we are not able to identify individually who of our participants was treated and who was not: the local district medical officer and Neglected Tropical Diseases (NTD) coordinator, who supervised the drug distribution in Kyela district, reported coverages of 60.8% in October 2009 and 68.2% in 2010 (Mrs. Masawe, personal communication). However, very few participants of the SOLF study were aware of the treatment program against LF, when asked about their participation. Therefore, firm assumptions about efficacy, i.e. the effect of antifilarial treatment on CFA prevalence and intensity under ideal conditions cannot be made. Instead our data are better suited to describe the effectiveness of MDA under real-life conditions. Furthermore, information on CD4 count and antiretroviral treatment status of HIV infected individuals would have helped to refine our analysis, but this information was not collected.

The Og4C3 antibody is supposed to specifically recognise *Wuchereria bancrofti* antigen with no relevant cross-reaction to *Onchocerca volvulus*, *Brugia spp*., *Mansonella*, *Dracunculus medinensis*, *Ascaris lumbricoides* or *Strongyloides stercoralis*. In spite of this, cross reactivity with *Loa* microfilariae has been found in another test (Binax Now Filariasis Immunochromatographic Test, Alere, Scarborough, ME, USA), which detects the same antigen as the Trop Bio ELISA [29]. However, there is no significant reported disease burden of Loiasis in our study area [30].

### Conclusion

In an area with high prevalence of and no previous treatment against LF we investigated the potential association of HIV and LF infection. When adjusting for age we found similar CFA prevalence and intensities in HIV-positive and negative participants. After two rounds of treatment a significant reduction in CFA prevalence and intensity was demonstrated, which was more pronounced in the HIV-positive compared to HIV-negative participants. Hence, HIV co-infection does not seem to negatively affect antifilarial treatment.

## References

[1] Hotez PJ, Kamath A. Neglected tropical diseases in sub-saharan Africa: review of their prevalence, distribution, and disease burden. PLoS Negl Trop Dis. 2009;3(8):e412 Epub 2009/08/27. 10.1371/journal.pntd.0000412 19707588 PMC2727001

[2] Simonsen PE, Magesa SM, Dunyo SK, Malecela-Lazaro MN, Michael E. The effect of single dose ivermectin alone or in combination with albendazole on Wuchereria bancrofti infection in primary school children in Tanzania. Trans R Soc Trop Med Hyg. 2004;98(8):462–72. 10.1016/j.trstmh.2003.12.005 . 15186934

[3] WHO. Global Programme to eliminate lymphatic filariasis: progress report on mass drug administration, 2010. Releve epidemiologique hebdomadaire / Section d'hygiene du Secretariat de la Societe des Nations = Weekly epidemiological record / Health Section of the Secretariat of the League of Nations. 2011;86(35):377–88. . 21887884

[4] Dreyer G, Addiss D, Noroes J, Amaral F, Rocha A, Coutinho A. Ultrasonographic assessment of the adulticidal efficacy of repeat high-dose ivermectin in bancroftian filariasis. Trop Med Int Health. 1996;1(4):427–32. . 8765448 10.1046/j.1365-3156.1996.d01-79.x

[5] Weil GJ, Ramzy RM, Chandrashekar R, Gad AM, Lowrie RC Jr, Faris R. Parasite antigenemia without microfilaremia in bancroftian filariasis. Am J Trop Med Hyg. 1996;55(3):333–7. . 8842125 10.4269/ajtmh.1996.55.333

[6] Dunyo SK, Nkrumah FK, Simonsen PE. Single-dose treatment of Wuchereria bancrofti infections with ivermectin and albendazole alone or in combination: evaluation of the potential for control at 12 months after treatment. Trans R Soc Trop Med Hyg. 2000;94(4):437–43. . 11127253 10.1016/s0035-9203(00)90135-4

[7] Dunyo SK, Nkrumah FK, Simonsen PE. A randomized double-blind placebo-controlled field trial of ivermectin and albendazole alone and in combination for the treatment of lymphatic filariasis in Ghana. Trans R Soc Trop Med Hyg. 2000;94(2):205–11. . 10897370 10.1016/s0035-9203(00)90278-5

[8] Simonsen PE, Pedersen EM, Rwegoshora RT, Malecela MN, Derua YA, Magesa SM. Lymphatic filariasis control in Tanzania: effect of repeated mass drug administration with ivermectin and albendazole on infection and transmission. PLoS Negl Trop Dis. 2010;4(6):e696 10.1371/journal.pntd.0000696 20532226 PMC2879369

[9] Msisha WM, Kapiga SH, Earls FJ, Subramanian SV. Place matters: multilevel investigation of HIV distribution in Tanzania. AIDS. 2008;22(6):741–8. 10.1097/QAD.0b013e3282f3947f 18356604 PMC2789284

[10] Tanzania NIMR-. Tanzania HIV/AIDS Malaria Indicator Survey (2011/2012) [01.02.1015].

[11] Layer EH, Kennedy CE, Beckham SW, Mbwambo JK, Likindikoki S, Davis WW, et al Multi-level factors affecting entry into and engagement in the HIV continuum of care in Iringa, Tanzania. PLoS One. 2014;9(8):e104961 10.1371/journal.pone.0104961 25119665 PMC4138017

[12] Layer EH, Brahmbhatt H, Beckham SW, Ntogwisangu J, Mwampashi A, Davis WW, et al "I pray that they accept me without scolding:" experiences with disengagement and re-engagement in HIV care and treatment services in Tanzania. AIDS Patient Care STDS. 2014;28(9):483–8. 10.1089/apc.2014.0077 . 25093247

[13] Hoerauf A, Pfarr K, Mand S, Debrah AY, Specht S. Filariasis in Africa—treatment challenges and prospects. Clin Microbiol Infect. 2011;17(7):977–85. Epub 2011/07/05. 10.1111/j.1469-0691.2011.03586.x . 21722251

[14] Ramaiah KD, Ottesen EA. Progress and impact of 13 years of the global programme to eliminate lymphatic filariasis on reducing the burden of filarial disease. PLoS Negl Trop Dis. 2014;8(11):e3319 10.1371/journal.pntd.0003319 25412180 PMC4239120

[15] Hotez PJ, Alvarado M, Basanez MG, Bolliger I, Bourne R, Boussinesq M, et al The global burden of disease study 2010: interpretation and implications for the neglected tropical diseases. PLoS Negl Trop Dis. 2014;8(7):e2865 10.1371/journal.pntd.0002865 25058013 PMC4109880

[16] Simonsen PE, Derua YA, Kisinza WN, Magesa SM, Malecela MN, Pedersen EM. Lymphatic filariasis control in Tanzania: effect of six rounds of mass drug administration with ivermectin and albendazole on infection and transmission. BMC Infect Dis. 2013;13:335 Epub 2013/07/23. 1471-2334-13-335 [pii] 10.1186/1471-2334-13-335 23870103 PMC3723586

[17] Simonsen PE, Magesa SM, Derua YA, Rwegoshora RT, Malecela MN, Pedersen EM. Monitoring lymphatic filariasis control in Tanzania: effect of repeated mass drug administration on circulating filarial antigen prevalence in young schoolchildren. Int Health. 2011;3(3):182–7. Epub 2011/09/01. j.inhe.2011.06.009 [pii] 10.1016/j.inhe.2011.06.009 . 24038368

[18] Talaat KR, Kumarasamy N, Swaminathan S, Gopinath R, Nutman TB. Filarial/human immunodeficiency virus coinfection in urban southern India. Am J Trop Med Hyg. 2008;79(4):558–60. Epub 2008/10/09. 79/4/558 [pii]. 18840744 PMC2596056

[19] Nielsen NO, Friis H, Magnussen P, Krarup H, Magesa S, Simonsen PE. Co-infection with subclinical HIV and Wuchereria bancrofti, and the role of malaria and hookworms, in adult Tanzanians: infection intensities, CD4/CD8 counts and cytokine responses. Trans R Soc Trop Med Hyg. 2007;101(6):602–12. Epub 2007/03/31. S0035-9203(07)00034-X [pii] 10.1016/j.trstmh.2007.02.009 . 17395223

[20] Nielsen NO, Simonsen PE, Magnussen P, Magesa S, Friis H. Cross-sectional relationship between HIV, lymphatic filariasis and other parasitic infections in adults in coastal northeastern Tanzania. Trans R Soc Trop Med Hyg. 2006;100(6):543–50. 10.1016/j.trstmh.2005.08.016 . 16324731

[21] Tafatatha T, Taegtmeyer M, Ngwira B, Phiri A, Kondowe M, Piston W, et al Human Immunodeficiency Virus, Antiretroviral Therapy and Markers of Lymphatic Filariasis Infection: A Cross-sectional Study in Rural Northern Malawi. PLoS Negl Trop Dis. 2015;9(6):e0003825 10.1371/journal.pntd.0003825 26042839 PMC4456405

[22] Talaat KR, Babu S, Menon P, Kumarasamy N, Sharma J, Arumugam J, et al Treatment of W. bancrofti (Wb) in HIV/Wb coinfections in South India. PLoS Negl Trop Dis. 2015;9(3):e0003622 10.1371/journal.pntd.0003622 25793933 PMC4368731

[23] Kroidl I, Clowes P, Mwalongo W, Maganga L, Maboko L, Kroidl AL, et al Low specificity of determine HIV1/2 RDT using whole blood in south west Tanzania. PLoS One. 2012;7(6):e39529 Epub 2012/07/07. 10.1371/journal.pone.0039529 PONE-D-11-13142 [pii]. 22768086 PMC3387183

[24] Rocha A, Addiss D, Ribeiro ME, Noroes J, Baliza M, Medeiros Z, et al Evaluation of the Og4C3 ELISA in Wuchereria bancrofti infection: infected persons with undetectable or ultra-low microfilarial densities. Trop Med Int Health. 1996;1(6):859–64. . 8980602 10.1111/j.1365-3156.1996.tb00123.x

[25] Chanteau S, Moulia-Pelat JP, Glaziou P, Nguyen NL, Luquiaud P, Plichart C, et al Og4C3 circulating antigen: a marker of infection and adult worm burden in Wuchereria bancrofti filariasis. J Infect Dis. 1994;170(1):247–50. . 8014511 10.1093/infdis/170.1.247

[26] Lammie PJ, Reiss MD, Dimock KA, Streit TG, Roberts JM, Eberhard ML. Longitudinal analysis of the development of filarial infection and antifilarial immunity in a cohort of Haitian children. Am J Trop Med Hyg. 1998;59(2):217–21. . 9715935 10.4269/ajtmh.1998.59.217

[27] Simonsen PE, Meyrowitsch DW, Mukoko DA, Pedersen EM, Malecela-Lazaro MN, Rwegoshora RT, et al The effect of repeated half-yearly diethylcarbamazine mass treatment on Wuchereria bancrofti infection and transmission in two East African communities with different levels of endemicity. Am J Trop Med Hyg. 2004;70(1):63–71. . 14971700

[28] Tafatatha TT, Ngwira BM, Taegtmeyer M, Phiri AJ, Wilson TP, Banda LG, et al Randomised controlled clinical trial of increased dose and frequency of albendazole and ivermectin on Wuchereria bancrofti microfilarial clearance in northern Malawi. Trans R Soc Trop Med Hyg. 2015;109(6):393–9. 10.1093/trstmh/trv027 . 25877874

[29] Wanji S, Amvongo-Adjia N, Koudou B, Njouendou AJ, Chounna Ndongmo PW, Kengne-Ouafo JA, et al Cross-Reactivity of Filariais ICT Cards in Areas of Contrasting Endemicity of Loa loa and Mansonella perstans in Cameroon: Implications for Shrinking of the Lymphatic Filariasis Map in the Central African Region. PLoS Negl Trop Dis. 2015;9(11):e0004184 10.1371/journal.pntd.0004184 26544042 PMC4636288

[30] Zoure HG, Wanji S, Noma M, Amazigo UV, Diggle PJ, Tekle AH, et al The geographic distribution of Loa loa in Africa: results of large-scale implementation of the Rapid Assessment Procedure for Loiasis (RAPLOA). PLoS Negl Trop Dis. 2011;5(6):e1210 10.1371/journal.pntd.0001210 21738809 PMC3125145

