# Correction: Prevalence of Lymphatic Filariasis and Treatment Effectiveness of Albendazole/ Ivermectin in Individuals with HIV Co-infection in Southwest-Tanzania

**DOI:** 10.1371/journal.pntd.0004967

**Published:** 2016-08-26

**Authors:** Inge Kroidl, Elmar Saathoff, Lucas Maganga, Petra Clowes, Leonard Maboko, Achim Hoerauf, Williams H. Makunde, Antelmo Haule, Prisca Mviombo, Bettina Pitter, Neema Mgeni, Joseph Mabuye, Dickens Kowuor, Upendo Mwingira, Mwelecele N. Malecela, Thomas Löscher, Michael Hoelscher

The second author's name is spelled incorrectly. The correct name is: Elmar Saathoff. The correct citation is Kroidl I, Saathoff E, Maganga L, Clowes P, Maboko L, et al. (2016) Prevalence of Lymphatic Filariasis and Treatment Effectiveness of Albendazole/ Ivermectin in Individuals with HIV Co-infection in Southwest-Tanzania. PLoS Negl Trop Dis 10(4): e0004618. doi: 10.1371/journal.pntd.0004618
